# The intrinsic structure and interrelations of tea culture constructed from tea-related toponym texts: Evidence from China

**DOI:** 10.1371/journal.pone.0347109

**Published:** 2026-04-17

**Authors:** Yiru Xu, Rong Wang, Hongqi Wu

**Affiliations:** 1 College of Liberal Arts, Jinan University, Guangzhou, China; 2 College of Tourism & Landscape Architecture, Guilin University of Technology, Guilin, China; University of South Carolina, UNITED STATES OF AMERICA

## Abstract

Tea culture is an important part of the regional culture and intangible cultural heritage that is valued and favored worldwide. The connotations of tea culture are mapped and precipitated in toponyms, which significantly influence the naming and renaming of said toponyms. However, rigorous research integrating tea culture and geographical naming systems is still premature, with a robust analytical framework yet to be established. This study explores China’s Hunan Province as the main research area, adopts the grounded theory method to explore the connotations of tea culture in tea-related toponym texts, and examines in depth the structure of tea culture as well as its internal relationships. The study notes that the more profound the tea culture, the more tea-related toponyms. Evidently, tea culture and toponyms are related. The results show that, on the one hand, the structure of tea culture can be deconstructed into six elements, namely tea tree, tea custom, tea industry, tea activity, tea polity, and tea shape. The structural inheritance and development of tea culture are the result of the interaction of various elements. On the other hand, tea culture’s internal structure comprises two interactive unit systems. From the overall-unit perspective, the natural environment and cultural atmosphere interact. The tea tree is the core of the natural environment, while the cultural atmosphere is jointly created by tea custom, tea industry, tea activity, tea polity, and tea shape. From the perspective of internal structural units, the six elements of tea culture’s structure interact to form an “effect-feedback” mutual influence pattern. This study systematically elucidates the structure of tea culture and interactions between its constituent elements. Such an analysis is crucial for a deeper understanding of local tea culture’s structural inheritance mechanisms. This study provides theoretical support and practical guidance for the development of tea-related industries.

## Introduction

As an important intangible cultural heritage, tea culture has long played a key role in social etiquette, economic development, and cultural exchange worldwide [1]. China, Britain, Japan, South Korea, India, Turkey, and other countries have developed unique tea-drinking traditions, reflecting diverse historical paths and local values [2,3]. In recent decades, the cultural, economic, and social value of tea has been increasingly recognized internationally [4]. International organizations and governments have also gradually reinforced their efforts to protect and promote tea culture [5]. In 2022, the United Nations Educational, Scientific and Cultural Organization (UNESCO) included two tea-related techniques in the Representative List of the Intangible Cultural Heritage of Humanity: “Traditional tea processing techniques and associated social practices in China,” and Azerbaijan and Turkey’s “Culture of Çay (tea), a symbol of identity, hospitality and social interaction.” Over the years, the Food and Agriculture Organization of the United Nations (FAO) has listed several tea-related agricultural heritage systems under its Globally Important Agricultural Heritage Systems (GIAHS). These include the Pu’er Traditional Tea Agrosystem, the Jasmine and Tea Culture System of Fuzhou City, the Traditional Wasabi Cultivation in Shizuoka in Japan, and the Traditional Hadong Tea Agrosystem in Hwagae-myeon in South Korea. Simultaneously, amid cultural integration and development, tea culture is being increasingly explored, protected, and inherited worldwide. However, various regions face problems regarding the protection and inheritance of tea culture, such as unclear connotations and insufficient explanatory power of theoretical frameworks [6,7]. Studying the structural elements of tea culture and their internal relationships deepens understanding of the said culture’s connotations and provides theoretical and practical support for the protection and inheritance of tea cultural heritage, as well as for innovation in the tea industry.

China is the birthplace of tea culture [8] and ranks first worldwide in tea cultivation and production [9]. Over thousands of years of evolution, Chinese tea culture has transcended agricultural techniques and dietary habits, developing into a complex cultural system that integrates material practices, social etiquette, and spiritual values [10–12]. This system exhibits a significant hierarchical structure. At the material level, it embodies agricultural wisdom, ranging from wild gathering to domestication and standardized production. At the behavioral level, it is internalized into daily customs, including consumption, medicinal use, health preservation, and hospitality [13,14]. At the spiritual level, it integrates Confucianism, Buddhism, and Taoism. This fusion forms a cultural character and philosophical height centered on the “Unity of Heaven and Humanity” [15]. Furthermore, tea culture is highly open and fluid, allowing it to take root locally while spreading globally via the Silk Road, Wanli Cha Dao, and other routes [16]. Through interaction with different civilizations, such as Britain, Russia, Japan, South Korea, India, and Turkey, tea culture has demonstrated strong adaptability and regenerative capacity [17,18], evolving into diverse regional forms. This evolutionary process, which transcends time and space and integrates multiple dimensions, has endowed tea culture with extremely rich connotations; however, it has also produced a complex internal structure that urgently needs to be deconstructed and clarified from a systematic perspective.

In this study, toponyms, as important carriers of regional culture, are treated as having a potential relationship with tea culture. For example, in China’s provincial units, regions with developed tea culture have formed numerous tea-related toponyms. The toponym texts, such as historical evolution, definitions, and naming reasons, reveal rich connotations of tea culture. For example, Chaxian River, a residential area in Dong’an County, Yongzhou City, Hunan Province, derives its name from legends related to tea (cha). Tradition holds that a young girl picking tea drowned in the river and became a deity, symbolizing tea’s high status in local life. Similarly, the name Shangchachongwei, a residential area in Xiangtan County, Xiangtan City, Hunan Province, originates from local tea (cha) customs. In wealthy families, when guests arrived, people would shout “Shangcha” (bring tea), instructing servants to serve it—a vivid reflection of tea as a symbol of etiquette. Evidently, tea-related toponym texts are crucial clues for constructing the structure of tea culture and analyzing the relationships and interactions among its internal elements, which is precisely what the current study delivers. This approach has two distinct advantages. First, it mitigates data subjectivity and memory bias. Second, the substantial sample size of toponyms ensures macroregional coverage, enabling the abstraction of concrete cultural structures from a panoramic perspective.

Therefore, drawing on the “Four-Tier Theory” in cultural theory [19], this study constructs a structural model of tea culture encompassing material, institutional, customary, ideological, and value dimensions. Textual materials on tea-related toponyms were collected and analyzed using grounded theory to refine the core concepts and categories of tea culture and develop a systematic framework. Based on this framework, internal connections among elements were analyzed to clarify theoretical perspectives and reveal tea culture’s essential connotations and formation mechanisms. The results indicate that, on the one hand, the structure of tea culture can be deconstructed into six elements, namely tea tree, tea custom, tea industry, tea activity, tea polity, and tea shape. The structural inheritance and development of tea culture result from the interaction among these elements. On the other hand, the internal structure of tea culture comprises two interactive unit systems. From the overall-unit perspective, the natural environment and cultural atmosphere interact. The tea tree element is the core of the natural environment, while tea custom, tea industry, tea activity, tea polity, and tea shape jointly form the cultural atmosphere. From the perspective of internal structural units, the six elements of the structure of tea culture interact to form an “effect-feedback” mutual influence pattern. This study aims to construct a tea culture structural system and clarify the internal relationships within the structure of tea culture, with innovations in research perspectives, theoretical systems, and interdisciplinary integration. In doing so, it enhances the understanding of the connotations of tea culture and fully leverages the symbolic value of tea in social etiquette, economic development, and cultural interactions.

## Literature review

### Tea culture

Tea, originating in ancient China and later adopted by Japan, has become a beverage of choice in many countries, including the UK, Russia, and Kuwait [7]. With a vast and influential history, tea culture has long attracted scholarly attention worldwide. Existing research has mainly explored tea culture’s multiple dimensions, origins, history, spirituality, social functions, and contemporary practices, showing diverse regional characteristics and research paths.

The origin and history of tea culture are important research topics. Research to date has primarily investigated the formation and evolution of tea culture from a macro perspective, such as history and culture, including core topics such as the origin and development of tea-drinking customs [20] and the formation and dissemination of the spirit of the tea ceremony [21]. Tea culture is not only considered a cultural concept rooted in tradition [22], but also carries historical heritage and spiritual connotations [23]. Its spiritual core is reflected through tea rituals, customs, and classics, which collectively form the core system of Chinese tea culture [21,24]. Tea has served as a symbolic medium in various important ceremonies and occasions [25]. For instance, in traditional Chinese weddings, tea symbolizes marital commitment and serves as a medium in key ceremonies, reaffirming kinship and social relationships [26]. Tea’s economic function, especially its integration into tourism development, has also attracted extensive scholarly discussion. Su Mingming argued that tea, as an agricultural product with strong sociocultural values, can be integrated into tourism to promote sustainable community livelihoods [27]. Moreover, through in-depth interviews, field visits, and participatory observations, Shen explored how integrating tea and tourism can revitalize ancient villages [28]. Via experimental research, Qi verified that tea culture can be linked to the development of the recreational industry [29]. Using East Frisian tea culture as a case study, Bohne explored its regional uniqueness and impact on tourism [30]. He argued that UNESCO-recognized intangible heritage, such as East Frisian tea culture, Turkish tea culture, and culinary mapping (e.g., Georgian tea routes), successfully links European heritage awareness, tourism management, and sustainable development [30]. Additionally, the inheritance, innovation, and sustainable development of tea culture have garnered extensive attention. Scholars have explored the contemporary value and practical pathways of tea culture from multiple perspectives, including cultural heritage protection [31], educational practice [32], design application [33], and cultural diplomacy [34].

The characteristics of tea culture differ by country. Related research has also shown diverse regional characteristics and research paths. The tea cultures of China, Japan, South Korea, Britain, Sri Lanka, Turkey, Ukraine, and Russia have sparked extensive discussion [35]. The study of Chinese tea culture began early, with Li Xiusong arguing that Chinese tea culture encompasses nearly every aspect of life, including physical and mental dimensions [12]. Research concerning Chinese tea culture focuses on how tea marketing and tea drinking interact with political, economic, and cultural life. Japanese tea ceremonies are closely related to the rituals, material culture, and political authority of early Japan, and were institutionalized into a comprehensive cultural system during the shogunate period [36]. Kim et al. identified the complex layers of Korean tea culture, which include tea texts, sets, ceremonies, and places, all evolving over time with distinct historical characteristics. Based on these elements, they established a semantic wiki for Korean tea heritage [37]. Whitfield et al. explored the authentic identity and social positioning of British tea culture through literary works [38]. Further, Botejue comprehensively analyzed Sri Lankan tea culture from ethnographic and archaeological perspectives, examining material culture, language, behavior, and political influences associated with tea traditions [39].

### Toponyms and tea culture

In cultural geography, toponyms reflect human cognition and the naming of natural and human environments [40]. Toponyms are not only identifiers of geographical entities, but also cultural symbols carrying history, memory, and local identity [41]. International research on toponyms is expanding across multiple dimensions. Some research has explored the spatial distribution and evolution of toponym cultural landscapes, attempting to uncover the historical processes of human-land interactions. Meanwhile, toponyms are increasingly valued for preserving cultural heritage, shaping local identities, promoting culture, and supporting economic development.

Toponyms have distinct temporal and spatial attributes, with their diachronic evolution and spatial distribution constituting key research areas. Guo et al. analyzed traditional village names in the Yulong Kashi River Basin, constructing a mapping system based on semantics, spatial distribution, historical evolution, and influencing factors to support the protection of international toponym heritage [41]. Many scholars have rigorously explored the spatial distribution of toponyms and their formation mechanisms under static conditions [42,43]. Some scholars have also assessed the spatiotemporal evolutionary characteristics and causes of the dynamically changing toponym cultural landscape [44,45]. As an internationally recognized form of intangible cultural heritage, toponyms embody local politics, economy, and culture, serving as important clues for regional research. Toponym cultural heritage holds significance for protection and inheritance [46], with toponyms helping to understand a place’s history, memory, and culture [47], all of which are closely related to the local environment. The diversity of toponyms reflects the human-land relationship, while also influencing the development of this relationship [48].

The temporal and spatial attributes of toponyms and their status as a form of intangible cultural heritage make them an important entry point for exploring local culture. Regions with developed tea culture exhibit a clear intrinsic connection between toponyms and the said culture. Tea culture is rich in connotation, long-standing, and deeply rooted in regional characteristics [30]. As tea culture has evolved, these cultural elements have been reflected in and incorporated into toponyms, profoundly influencing place naming and renaming. Many toponyms are rich in tea culture, documenting unique local tea varieties, tea-related activities, and tea-drinking customs. Therefore, although current research has largely overlooked this domain, exploring tea culture through the lens of toponyms‌ is feasible. Existing studies have primarily explored tea’s cultural connotations within tea-related toponyms, examining their historical development from perspectives such as etymology [49], interpretation, and evolution [50]. Interpreting tea-related toponyms, such as Zhushan [51], Pu’er Simao [52], and Jianou Beiyuan, remains a key research direction. Conducting more of these studies would not only reveal the connection between toponyms and tea culture events, but also reconstruct the historical context of the development of the local tea industry. Existing research has primarily been limited to case interpretation or lexical verification and lacks systematic review and theoretical integration of tea culture-related toponyms. Comprehensive research integrating tea culture and geographical naming systems is still in its infancy, and a robust analytical framework has yet to be established.

### Research gap

Existing studies have largely adopted classical paradigms in cultural sociology, conceptualizing the structure of tea culture as either a “material-spiritual” dualistic structure or a “material-institutional-spiritual-behavioral” four-layer structure. Regarding the former, Wang proposed, in relation to Chinese tea culture, that tea culture itself ingeniously integrates material and spiritual cultures [53]. Regarding the latter, Yao Guokun argued that the manifestations of tea culture can be grouped into four aspects: material, institutional, spiritual, and behavioral [54].

Existing research concerning the deconstruction of tea culture’s connotations is primarily based on a priori top-down deductive theoretical frameworks. This macro perspective outlines the overall contours of tea culture; however, its explanatory power regarding the culture’s structural elements and the relationships between them is limited. The above-mentioned perspective often overlooks industry-driven factors and cognitive projections, failing to explain the mutual influence of tea culture’s various structural elements. Against this backdrop, toponym texts, as carriers of local culture, possess stability in temporal transmission and wide spatial distribution, thus providing a “universal key” for comprehensive research on tea culture in different regions. Notably, at the methodological level, recent studies have applied grounded theory, as a bottom-up qualitative research method, in fields such as cultural heritage value mining [55] and local memory deconstruction and its impact [56]. The aforementioned demonstrates the significant advantages of this method for processing unstructured text data and extracting localized cultural theories. However, although toponym texts contain rich cultural information and it has been proven that grounded theory is applicable to research on the construction of local cultural structures, no research has yet combined the two and systematically applied them to the construction of tea culture structures.

In light of this, grounded in toponym texts, the current study extracts tea culture elements to build a more systematic and detailed structure, analyzing the interrelationships among those elements. It attempts to explore the internal logic of the structural inheritance of tea cultural heritage from a dynamic standpoint, providing a new perspective that explains the mechanism behind the interaction between the natural environment and the cultural atmosphere.

## Materials and methods

### Research area

Considering the practical needs of this study, the research area must meet two main criteria. First, the relevant tea culture should be long-standing and possess a profound heritage. Second, it should be extensively influential, hold significant status in China, and be widely recognized worldwide. After comprehensively evaluating both the historical depth and global influence of tea culture, Hunan Province in China was chosen as the primary research region.

From a historical perspective, Hunan Province’s tea culture demonstrates temporal integrity and representativeness in its transmission and development. The legend of Shennong “using tea to detoxify” has historically linked Hunan with the origin of tea culture, elevating it to a sacred cultural source. More importantly, the physical tea artifact known as “Jiasi,” unearthed from the Mawangdui Tombs of the Han Dynasty in Changsha, Hunan, stands as the world’s oldest discovered tea specimen. This archaeological evidence definitively proves that tea-drinking customs existed during the Western Han period, indicating that the history of tea culture stretches back over 2,100 years. From Lu Yu’s Tang Dynasty classic *The Classic of Tea*, which documents “Li Yang tea in Li Prefecture,” to the Ming and Qing dynasties, it is evident that Anhua Dark Tea became official tea and was exported to frontier regions and overseas markets. The development of tea culture in Hunan is clear and uninterrupted. This continuous evolution provides a rich and enduring case study for diachronic research.

Regarding influence, Hunan Province is among the core tea-producing provinces in China’s tea industry landscape, having produced globally impactful tea varieties and brands. Hunan Province has nurtured iconic tea categories such as Anhua Dark Tea and Junshanyinzhen Tea. Among these, Anhua Dark Tea was a key commodity along “the ancient tea horse trail,” exerting cross-border influence by reaching Mongolia, Russia, and other regions. Ultimately, Anhua Dark Tea from Hunan Province became an internationally renowned brand.

Hunan’s developed tea culture has led to the formation of numerous residential tea-related toponyms. According to preliminary statistics, there are 6,151 such toponyms in Hunan Province, making it the province with the highest number of tea-related toponyms in China. Therefore, selecting Hunan Province as the primary research area fully meets the current project’s needs in building structures of tea culture based on toponym texts. This ensures that the study’s conclusions are representative and academically valuable.

### Methodology selection

This study builds a tea culture structure using toponym texts and analyzes the interrelationships among its elements. A constructivist grounded theory research method was adopted for several reasons.

First, this methodological selection was determined by the significant social attributes of the toponym text data. Charmaz’s constructivist grounded theory acknowledges that data and analysis emerge from researchers’ interactions with texts [57,58] and is applicable to situations involving multiple interacting elements. It has been widely applied in studies of local culture and ethnic behavior [59–62]. Toponyms are not merely geographical labels but cultural symbols constructed within specific historical and social contexts [63,64]. Constructivist grounded theory therefore encourages the interpretation of legends, customs, and historical contexts behind toponyms, which is crucial for uncovering the profound cultural connotations condensed in toponym texts.

Second, the constructivist grounded theory research methodology aligns with the nature of this study’s theoretical exploration. The projection of tea culture using toponyms is highly metaphorical and complex. However, the academic community has not yet formed a mature theoretical framework for presupposing coding categories. Grounded theory uses a bottom-up inductive logic, making it suitable for researchers to conduct exploratory research from scratch [65]. Therefore, adopting constructivist grounded theory to directly extract concepts and categories from toponym texts, thereby constructing a more specific tea culture structure that fits the local context, is scientifically appropriate as a research process.

Third, constructivist grounded theory has unique theoretical explanatory power in revealing “intrinsic interrelations” between elements [66]. While attempting to deconstruct the structural elements of tea culture, this study is dedicated to answering questions regarding the interrelationships among the said culture’s elements. Constructivist grounded theory, through steps such as “theory coding” and “storyline construction,” enables researchers to move beyond static topic descriptions and examine the logical relationships between core categories, thus achieving a transition from “phenomenon description” to “theory construction.” Constructivist grounded theory offers a significant advantage in terms of research depth.

### Data collection and cleaning

This study adopted a “full population sampling” strategy for the specific type of data assessed, namely toponym texts [67], which is conducive to eliminating sampling errors from a macro perspective [68]. The goal behind this was to ensure the structural integrity of the theoretical construction and effectively avoid the omission of key marginal nodes in specific regional cultures due to sampling bias [69]. The primary sources of toponym text data for this study included the National Database for Geographical Names of China (https://dmfw.mca.gov.cn/, accessed September 30, 2025, hereafter referred to as the Information Database), GPS Toolkits (https://web.gpstool.com/index, accessed September 30, 2025), Baidu Maps (https://map.baidu.com/, accessed September 30, 2025), and Map Location (https://maplocation.sjfkai.com/, accessed September 30, 2025). First, data related to residential toponyms containing “cha” (tea) in Hunan Province were obtained through the Information Database by entering the character “cha” (tea) into the website’s search box, setting the administrative division to Hunan Province, and limiting the toponym category to residential places. This yielded 6,151 initial toponyms, from which information such as addresses, origins, meanings, and historical evolution was extracted to form the toponym text. Second, toponyms containing “cha” (tea) that explicitly refer to non-tea plants, such as “you cha” (camellia oleifera), “cha hua” (camellia japonica), or “cha zi” (camellia seed), were screened out, while data with broader “cha shu” (camellia sinensis) connotations were retained. The sample data were refined to focus on tea culture, resulting in 5,771 toponym data entries containing “cha” (tea).

Preliminary analysis revealed issues of authenticity, timeliness, duplication, and grouping of toponyms in the toponym data, which were addressed through corresponding measures. First, the accuracy and reliability of toponyms’ coordinates were used to verify authenticity. Specifically, all 5,771 toponym addresses were entered into the Map Location website, and their coordinate data, along with accuracy and reliability metrics, were batch-exported. Places with reliability values above 50 were retained. Table 1 shows the absolute precision of interpretation errors corresponding to reliability values. Second, the timeliness issue was addressed by comparing locations across multiple map websites. After comparison, if a toponym had been renamed or had disappeared but had not been updated in the Information Database, the corresponding data were deleted. Third, the issue of repeated toponyms in the same location, with similar origins, meanings, and historical evolution, was addressed. Selective deduplication was adopted; that is, entries with less information were deleted, and toponym data with more complete information were retained. Fourth, to address the issue of numerous natural villages divided into multiple groups of toponyms with the same proper name owing to the zoning management strategy, only the data of natural village names or the first group of toponyms were retained. Through preliminary statistics and secondary analysis, 3,607 valid toponyms were identified. The toponyms that reflect tea culture were defined and standardized as “tea-related toponyms.” After thorough organization, reading, and screening of the 3,607 tea-related toponyms, a 130,000-character analytical report was compiled. Notably, the Chinese pronunciation of “tea” is “cha.” Owing to the unique nature of toponyms, English expressions follow literal translations. Hence, all instances of tea in the analyzed toponyms were consistently presented as “cha” in this study.

**Table 1:** Credibility interpretation of the Maplocation website.

Trustworthiness values (≥)	100	90	80	75	70	60	50	40	30	25	20
**Absolute accuracy of analytical error (<)/10m**	2	5	10	20	30	50	100	200	500	800	1000

### Data processing

Based on the grounded theory process of “open coding, axial coding, and selective coding,” the specific manifestations of tea culture in Hunan’s tea-related toponym texts were explored, and the corresponding types were summarized. Because the collected textual data on tea-related toponyms were relatively scattered, conceptualizing and categorizing them did not pose a risk of losing authenticity, making the methodology feasible and reliable. This study implemented the principle of “progressive coding” during the coding phase, enhancing conceptualization through gradual data refinement and optimization. Conceptualization was undertaken to form initial concepts from the original texts of tea-related toponyms. Common factors among these initial concepts were extracted to form subcategories within the tea culture structure. These subcategories were further classified based on their correlational characteristics. Subcategories with strong logical relevance were summarized to extract main categories through progressive induction.

This study used the NVivo 11 (developed by QSR International) to code “tea-related toponym” texts in Hunan Province, China, ensuring a systematic and visual understanding of the coding process. First, data were imported and preprocessed. The Hunan Province “tea-related toponym” texts, which were manually cleaned during the previous “data collection and cleaning” process, were imported into the “Internals” folder of the software, and the “Sources” file library was established. This library was classified by the administrative division of prefecture-level cities. Second, node creation and open coding were performed, mainly by coding text data line by line through the “Nodes” function module. While reading the source texts, keywords and phrases related tea cultural implications were selected, and “Free Nodes” were created via the “Code” function, corresponding to the extraction of initial concepts in grounded theory. Subsequently, hierarchical construction and axial coding were performed. The NVivo directory tree structure was used for induction. In the “Nodes” view, parent-child relationships were established through drag-and-drop operations, merging “Free Nodes” into “Tree Nodes” to form a logical hierarchy from “initial concepts” to “sub-categories” and then to “main categories.” Finally, tea culture theory was constructed. Based on the summary of the parent-child node ideas using the “Memos” function, and referring to the visualization of the node relationship paths in the “Project Map” function, combined with the specific insights gained during the coding process, the “storyline” of the selective coding stage was organized and the theoretical model was constructed.

### Open coding

Open coding follows the logic of “defining phenomena to develop concepts,” involving word-by-word reading, organization, and analysis of the original text to achieve preliminary conceptualization. Taking the toponym Dachahu as an example, it originates from a sound change in Tujia. “Chahu” is a Tujia word meaning “good valley.” During the open coding phase, the most important task was to decompose the text word by word and extract initial tags such as “Tujia phonetic changes” and “conversion between different languages.” Comparative analysis revealed that this label has significant commonalities with labels such as “derived from a Miao language toponym” and “conversion between different languages” in “Chadong Zhai.” These all involve the conversion of minority language toponyms into tea-related toponyms through transliteration, translation, and refinement, reflecting tea culture’s potential influence on ethnic interaction, exchange, and integration. Therefore, these initial labels related to minority languages were clustered into the initial concept of “A_24_: The languages of ethnic minorities are translated into Chinese according to their pronunciation,” and finally summarized into the subcategory “B_7:_ Language and culture.”

This process ensured that each category was closely rooted in the details of the original text. During this process, original phrases reflecting tea culture were restructured and categorized into Hunan’s tea-related toponyms, eliminating redundant and personalized expressions while standardizing variations of synonymous terms. This resulted in 71 initial concepts denoted as A_n_, which were further consolidated into more targeted categories, yielding 21 subcategories represented by B_n_. Owing to space limitations, only partial results from the conceptualization and categorization processes are presented (Table 2).

**Table 2:** Examples of the initial concepts and subcategories under open coding.

Primitive statements (example)	Label extraction	Conceptualization (A_n_)	Subcategory (B_n_)
**Chashu Wanli:** It was named Chashu Wanli because there was a tea tree about 4 meters high in this place before.	A 4-meter tea tree	**A**_**2**_: There was a tall tea tree	**B**_**1**_: Lonely ancient tea trees
**Xicha Yuan:** Xicha Yuan is a piece of dry land where tea is grown and the land contract system is implemented. It is allocated to seven or eight groups. Since the early 1980s, houses have been built to form a residential area.	A piece of dry land; Grow tea	**A**_**4**_: Grow all kinds of tea	**B**_**2**_: Tea planting and cultivation
**Maoping Cun Chayuan Zu:** The group was named Chayuan Zu because it had a large tea plantation and was always rich in tea production.	The original large tea plantation; Rich in tea production	**A**_**9**_: Tea plantations in the historical period	**B**_**3**_: Tea tree plantations
**Xincha Ting:** There is a large stone-paved path in the middle of the courtyard, which passes through the middle of the pavilion. On the Dragon Boat Festival, someone brews tea, and it is named Xincha Ting.	Someone brews tea on the Dragon Boat Festival; The pavilion	**A**_**12**_: Local customs are related to tea	**B**_**4**_: Faith and custom
**Cha’an Li:** In the early years, there was a hermitage dedicated to the tea god, hence the name Cha’an Li.	A hermitage; Dedicated to the tea god	**A**_**14**_: A temple was built to worship the god of tea	
**Hongcha Zu of Qianzhen Village:** In 1982, the Hongtai Group was divided into groups. The adjacent tea plantations were named Hongcha Zu, symbolizing that people’s life is like spring tea leaves, red and lively.	Adjacent tea plantations; It signifies a prosperous life	**A**_**15**_: The good meaning of tea without worry	**B**_**5**_: Beautiful metaphor
**Chadou Pang:** Legend has it that a man surnamed Li lived by a hillside at the foot of Huangmao Mountain. He planted several tea seedlings, but while others withered, one cluster in the center grew larger than a blue plate. The kind-hearted old man and his neighbor were so compassionate that when his wife fell gravely ill, she received divine guidance from Guanyin Bodhisattva. She cured her illness with tea leaves, and this miraculous plant grew to produce tea that could cure all diseases. As this area became a crucial route between Longwan Bridge and Yueyang City and gained fame for its tea, it was affectionately called Chadou Pang.	Divine guidance from Guanyin Bodhisattva; Tea could cure all diseases; A crucial route	**A**_**21**_: The legend of tea curing diseases	**B**_**6**_: Allusions and legends
**Chaxian River:** Legend has it that long ago, a tea-picking girl went to the river in front of the village to wade across but drowned and was later deified, hence the name.	Tea-picking girl; The girl becomes immortal	**A**_**23**_: The story of the tea-picking girl	
**Dachahu:** “Da” is an adjective; “Cha Hu” is a Tujia word, and in the Tujia script, it is “Carhur.” “Cha” is a phonetic variation of another word with a similar pronunciation, meaning “good”; “Hu” is translated as “valley” or “ditch” in Chinese.	Tujia phonetic changes; Conversion between different languages	**A**_**24**_: The languages of ethnic minorities are translated into Chinese according to their pronunciation	**B**_**7**_: Language and culture
**Chadong Zhai:** The toponym “Chadong” originates from the Miao language. “Cha” refers to Han Chinese, while “dong” means a hollow or pit. In ancient times, this area was inhabited by Han Chinese, hence the name Chadong.	Derived from Miao language toponym; Conversion between different languages		
**Chayuan Ling:** It is said that this place was originally the camp of Huang Chao’s troops, so it was called Zaying Ling, and later it was renamed Chayuan Ling owing to phonetic similarity.	The place where troops set up camp; Phonetic similarity	**A**_**25**_: Dialect homophonic beautification	
**Chachang of Wuliping Village:** The settlement was named after its location near the former tea processing factory in Huzhu Bay.	Near the tea processing factory	**A**_**44**_: A tea factory was built to make tea	**B**_**12**_: Tea production
**Chawu of Nanshan Village:** There was a house here that specialized in frying tea leaves, hence the name “Chawu.” “Chawu” is a house used for frying tea leaves.	A house specialized in frying tea leaves	**A**_**46**_: There are tea processing places	
**Chayuan Po:** In the early days after liberation, this place was mainly used for tea picking and was named Chayuan Po.	Tea picking is the main agricultural activity	**A**_**47**_: The agricultural activity of picking tea	**B**_**13**_: Tea-picking activities
**Chadou Po:** Because many tea trees were planted on the hillside, tea farmers settled here, hence the name Chadou Po.	Many tea trees were planted; Tea farmers settled here	**A**_**48**_: Where tea farmers live	
**Guancha Yuan:** The name refers to a town or settlement that originally supplied tea to the imperial court. Because the tea grown here was once tribute tea, the area was called Guancha Yuan.	Originally supplied tea to the imperial court; Tribute tea	**A**_**56**_: During the historical period, tea was supplied to the imperial court	**B**_**16**_: Taxation and tribute
**Chajing Chong:** The toponym is derived from the name of a well. There is a well in the valley whose water is tea-colored, hence the name.	The well water was tea-colored; Derived toponym	**A**_**70**_: Some wells have tea-colored water	**B**_**21**_: Tea-colored soil or water

### Axial coding

Core axial coding builds upon open coding by systematically summarizing and abstracting logical connections between subcategories [70]. Through cluster analysis, it clarifies categorical relationships, minimizes overlap between categories, and identifies the “core axis” connecting subcategories to establish higher-level main categories. Based on this approach, repeated comparisons and clustering of 21 subcategories identified six generalized structural categories of tea culture, namely “tea tree,” “tea custom,” “tea industry,” “tea activity,” “tea polity,” and “tea shape,” denoted as C_n_ (Table 3).

**Table 3:** Main categories and corresponding subcategories.

number	fundamental category	Corresponding subcategory
**C**_**1**_	Tea tree	**B**_**1**_: Lonely ancient tea trees; **B**_**2**_: Tea planting and cultivation; **B**_**3**_: Tea tree plantations
**C**_**2**_	Tea custom	**B**_**4**_: Faith and custom; **B**_**5**_: Beautiful metaphor; **B**_**6**_: Allusions and legends; **B**_**7**_: Language and culture
**C**_**3**_	Tea industry	**B**_**8**_: Making tea-related utensils; **B**_**9**_: Tea dealer track; **B**_**10**_: Tea trading business; **B**_**11**_: Running teahouses; **B**_**12**_: Tea production
**C**_**4**_	Tea activity	**B**_**13**_: Tea-picking activities; **B**_**14**_: Tea drinking utensils; **B**_**15**_: Tea drinking activities
**C**_**5**_	Tea polity	**B**_**16**_: Taxation and tribute; **B**_**17**_: Posthouse role
**C**_**6**_	Tea shape	**B**_**18**_: Terrain like tea utensils; **B**_**19**_: Water tasting like tea; **B**_**20**_: Terrain like tea-leaf; **B**_**21**_: Tea-colored soil or water

### Selective coding

Selective coding refines core categories to a higher level of abstraction, forming more overarching and representative core categories. A “storyline” connects these core categories with others, further analyzing their relationships and constructing theoretical models. Code analysis revealed that tea cultural elements in Hunan’s tea-related toponyms involve the six above-mentioned structural categories.

**Fig 1:**
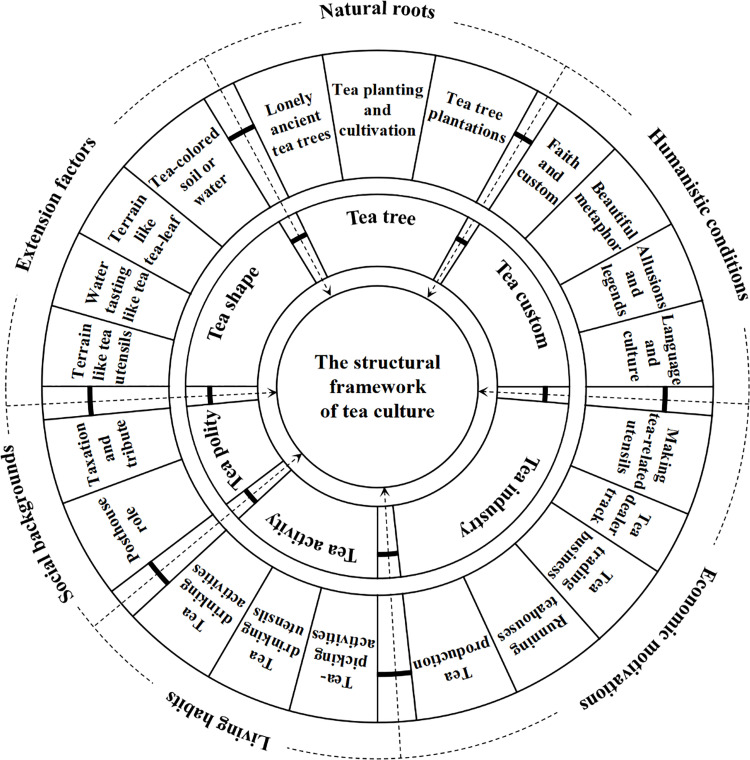
Tea culture structure “Lemon map”.

Further analysis of the relationships among these six main categories and their specific categories reveals a storyline that can be summarized as follows. From the perspective of toponyms exploring the structure and internal relationships of tea culture, tea tree serves as the natural roots, tea custom reflects the culture’s humanistic conditions, the tea industry represents economic motivations, tea activity mirrors living habits, tea polity describes social backgrounds, and tea shape, combined with external environments, serves as an extension of the cultural structure. Based on this “storyline,” a framework for Hunan’s tea culture structure was established (Fig 1).

The tea culture structure is a typical complex system. This study names the constructed tea culture structure diagram the “Lemon map” because its shape resembles the cross-section of a lemon, exhibiting multi-petal, multi-layered, and centrally convergent characteristics. The aim is to visualize the hierarchy and interrelationships of the various dimensions within the tea culture structure. As shown in Fig 1, the “Lemon map” visually demonstrates that the various categories of the tea culture structure are equal, parallel, and mutually permeable in geographical space, indicating that the tea culture structure is a “multi-dimensional unified” structural model.

Specifically, Fig 1 is divided into three main layers from the inside out. The core layer is “The structural framework of tea culture,” which is the central focus of this research. The middle layer includes the six above-mentioned core categories: tea tree, tea custom, tea industry, tea activity, tea polity, and tea shape. These are the main elements that constitute the tea culture structure. The outer layer is a refinement of the core categories and contains the specific content of the tea culture structure. Secondly, the arrows and boundary lines in the figure define the direction of action and specific division of tea culture’s structural elements. The arrows pointing to the core layer indicate the “centripetal effect” and “logical composition” of each core category and its subcategories on the structure of tea culture, showing that these dimensions jointly shape and support the composition and connotation of the tea culture structure. The categories are separated by dotted lines on the basis of solid lines, with the aim of expressing the mutual influence among different tea culture dimensions. These dimensions are composed of core categories and their subcategories. Finally, Fig 1 places the “Natural roots” at the top center and then arranges the “Humanistic conditions,” “Economic motivations,” “Living habits,” “Social backgrounds,” and “Extension factors” in a clockwise direction, forming a complete logical closed loop from the natural foundation to social construction.

### Theoretical saturation test

Theoretical saturation fundamentally refers to the complete exhaustion of categorical relationships through comparative analysis of new and original data [71], representing the ideal state in grounded theory and ensuring analytical reliability and stability [72]. This study first conducted code analysis to assess tea-related cultural connotations in tea-related toponyms outside Loudi City, Hunan Province, and produced preliminary results. Subsequently, structural saturation testing was applied to the cultural connotations within Loudi’s tea-related toponyms to refine the coding framework. Following identical coding procedures, all structural categories identified in Loudi’s tea-related toponyms exhibited repeated patterns, with no new categories or inter-category relationships emerging. This confirms that the coding results achieved theoretical saturation. Table 4 presents the three-level coding outcomes.

**Table 4:** Main categories and corresponding subcategories.

Core Scope	Subcategory	Initial Concepts
C_1_: Tea Tree	B_1_: Lonely ancient tea trees	A_1_: There are ancient tea trees that are many years old
		A_2_: There was a tall tea tree
		A_3_: The village above a tea tree
	B_2_: Tea planting and cultivation	A_4_: Grow all kinds of tea
		A_5_: The village used to grow tea in historical times
		A_6_: Artificially cultivated tea
		A_7_: Existing land for specialized tea
		A_8_: Abundant tea production
	B_3_: Tea tree plantations	A_9_: Tea plantations in the historical period
		A_10_: Tea trees grow in gardens
		A_11_: Wild tea plants are often grown in gardens
C_2_: Tea Custom	B_4_: Faith and custom	A_12_: Local customs are related to tea
		A_13_: Etiquette of drinking tea
		A_14_: A temple was built to worship the god of tea
	B_5_: Beautiful metaphor	A_15_: The good meaning of tea without worry
		A_16_: The beautiful meaning of tea fragrance
		A_17_: The good symbol of tea-leaf
	B_6_: Allusions and legends	A_18_: Legend of the magical tea tree
		A_19_: An allusion to the origin of tea culture
		A_20_: Famous people and tea legends
		A_21_: The legend of tea curing diseases
		A_22_: The legend of tea plantation
		A_23_: The story of the tea-picking girl
	B_7_: Language and culture	A_24_: The languages of ethnic minorities are translated into Chinese according to their pronunciation
		A_25_: Dialect homophonic beautification
		A_26_: Local names are special references
		A_27_: People with the surname “Cha” live here
C_3_: Tea Industry	B_8_: Making tea-related utensils	A_28_: Production of teacup clay
		A_29_: Some people know how to make tea trays
		A_30_: There are well-known masters of handmade tea utensils
	B_9_: Tea dealer track	A_31_: There used to be a dock for loading and unloading tea
		A_32_: There is a tea pavilion on the way to the customer
		A_33_: A pavilion built by a tea merchant
	B_10_: Tea trading business	A_34_: Wholesale or retail tea
		A_35_: There are tea shops for buying and selling
		A_36_: Tea houses that buy tea
		A_37_: The local economy depends on tea
		A_38_: The tea industry flourished in history
	B_11_: Running teahouses	A_39_: Open teahouses in local areas
		A_40_: Building a teahouse
		A_41_: Build a pavilion for opening a teahouse for pilgrims to drink tea
		A_42_: There are tea stalls
		A_43_: There was a teahouse
	B_12_: Tea production	A_44_: A tea factory was built to make tea
		A_45_: There are tea making companies
		A_46_: There are tea processing places
C_4_: Tea Activity	B_13_: Tea-picking activities	A_47_: The agricultural activity of picking tea
		A_48_: Where tea farmers live
		A_49_: Build a pavilion for people to pick tea and rest
	B_14_: Tea drinking utensils	A_50_: The residential area has two stone tea basins
	B_15_: Tea drinking activities	A_51_: Locals like drinking tea
		A_52_: There are tea tasting and tea culture propagating activities
		A_53_: Scholars drink tea and compose poems
		A_54_: There is a place to have tea and rest
		A_55_: Tea culture flourished locally
C_5_: Tea Polity	B_16_: Taxation and tribute	A_56_: During the historical period, tea was supplied to the imperial court
		A_57_: There was a customs duty on tea transported by water
	B_17_: Posthouse role	A_58_: There are places for pedestrians to drink tea and rest
		A_59_: A tea pavilion was built as a transportation station
C_6_: Tea Shape	B_18_: Terrain like tea utensils	A_60_: The terrain is similar to a tea-related utensil
		A_61_: The ground is similar to tea-related utensils
		A_62_: The terrain is similar to tea ware
	B_19_: Water tasting like tea	A_63_: The water from the well is as sweet as tea
		A_64_: Spring water is like tea
	B_20_: Terrain like tea-leaf	A_65_: The shape of the place resembles a tea leaf
		A_66_: The terrain resembles a tea stack
		A_67_: Terrain resembles a tea plantation
		A_68_: There are small beaches like Chaping
	B_21_: Tea-colored soil or water	A_69_: The soil is tea-colored
		A_70_: Some wells have tea-colored water
		A_71_: The stream is as green as tea

## Results

Through systematic collection, analysis, and coding of tea-related toponym texts, six core components of tea culture were identified: tea tree, tea custom, tea industry, tea activity, tea polity, and tea shape. The structural inheritance and evolution of tea culture emerge from the synergistic interaction of these elements. From a pattern perspective, the six tea culture elements exhibit a progressive shift from “natural materials” to “social and cultural elements.” From a mechanism perspective, tea culture reflected in toponyms forms a framework of “material identification-spiritual symbolism-industrial development-institutional governance-behavioral practice-cognitive projection.”

Among these components, tea tree, as a natural material entity, provides the intrinsic root for the formation of tea-related toponyms and reflects the close connection between the natural environment and tea culture. Tea custom showcases regional diversity through local traditions, linguistic practices, psychological customs, and folk narratives, thus demonstrating cultural richness. This indicates that tea culture is internalized into the collective memory and spiritual symbols of the region through its toponyms. Tea industry encompasses production, processing, and distribution, demonstrating the influence of tea culture on the economy, which in turn reflects the role of economic power in shaping the local cultural structure. Tea activity documents practices of daily behavior, such as tea harvesting and consumption, adding vitality to tea culture. Tea polity reflects the government’s policy orientation and management of the tea industry, serving as an important background for the development of tea culture. It also reflects how political forces shape local culture. Tea shape is a projection of the understanding of tea culture’s natural geographical features. Through the imagery of topography, landforms, and natural landscapes in tea-related toponyms, tea shape shows the integration of tea culture and the geographical environment. It is evident that the various elements of tea culture influence each other, completing the structural transformation of tea culture from “plant attributes” to “cultural attributes.”

### Tea tree elements as the natural roots

Tea tree elements are the natural roots of tea culture. Analysis shows that tea trees not only exist as plants, but also serve as key geographical references with significant locational and identifying functions, driving the formation and derivation of related toponyms through “salient features.” Tea tree includes three subcategories: lonely ancient tea trees, tea planting and cultivation, and tea tree plantation. These subcategories reveal the scale of tea culture’s evolution pattern in toponyms from “individual node” to “regional landscape.”

(1) Lonely ancient tea trees are toponyms in which the tea element originates from a unique single or clustered tea plant, or is exceptionally tall, historically significant, or culturally meaningful. It is obvious that a single tea tree with a special morphology or historical value often becomes the core coordinate of the micro-geographical space, reflecting a landmark at the individual scale. For instance, Chashu Wanli derived its name from a four-meter-tall tea tree that once grew there. Its essence lies in residents transforming significant biological characteristics into permanent spatial positioning symbols. (2) Tea planting and cultivation involve toponyms in which the tea element stems from local tea plantings, reflecting the definition of land attributes through human agricultural behavior and the functionalization of the behavioral scale. Verbs such as “planting,” “cultivating,” and “sowing” record changes in land use in a specific historical period. For example, the name “Chadouli” refers to numerous green tea trees planted during the Qing Dynasty. This shows that the toponym not only indicates the location, but also solidifies the historical memory of green tea cultivation since the Qing Dynasty, reflecting the mechanism by which production activities influence the toponym system. (3) Tea tree plantations include most residential tea-related toponyms, referring to places where existing or historical tea gardens serve as tea sources without direct agricultural involvement. This represents the large-scale spatial expansion of tea culture at the landscape scale. Toponyms such as Wangjia Chayuan and Chachongli demonstrate how natural tea forests or artificial tea gardens transform natural spaces into social spaces with ownership, resources, cultural, or landscape characteristics through the process of “fieldization.” These natural attributes of tea culture directly influence the naming of tea-related toponyms in Hunan Province and serve as the foundation for other cultural elements.

### Tea custom elements reflect the humanistic conditions

Tea custom elements reflect the humanistic conditions of tea culture. Their formation mechanism anchors tea-related social customs, beliefs, and language into spatial names through toponyms, enabling the intergenerational inheritance of local culture and memory. Through textual analysis, this study categorizes beliefs, auspicious, cultural, and linguistic features as tea-related customs. These elements collectively reflect the local humanistic conditions in Hunan’s tea-growing regions, including customs, traditions, language, psychology, and folk narratives. This reveals the deep structure of tea culture at the social, psychological, and spiritual levels.

(1) The subcategory “faith and custom” reflects local tea-related practices, drinking etiquette, and religious rituals. This shows that toponyms function as containers that transform instantaneous ritual activities into long-term cultural memory and realize the spatial solidification of local rituals and beliefs. The toponym Xincha Ting records the folk custom of offering tea during the Dragon Boat Festival, while Shangchachongwei solidifies the hospitality etiquette of “offering tea to guests” into a toponym. This mechanism makes abstract social norms more concrete in geographical entities. (2) The two subcategories “beautiful metaphor” and “allusions and legends” show that tea culture gives physical space a cultural narrative meaning beyond daily experience, reflecting the reconstruction of a sense of place through narrative. For example, the legend of the tea-picking fairy in the Chaxian River endows the place with sacredness and cultural depth through mythological narrative; Chaxiang Yuan reflects the emotional projection mechanism by which residents express their ideal of a life of “peaceful livelihoods and tea-scented leisure” through tea culture. (3) The subcategory “language and culture” provides strong evidence of the cross-ethnic spread of tea culture, representing linguistic acculturation through cross-cultural interactions. Analysis revealed that, in ethnic minority areas of western Hunan Province, there are many instances of words from the Dong, Miao, and Tujia languages being transliterated or refined into the character “cha” (tea). For example, the “cha” in “Chadong Zhai” actually means “Han Chinese” in the Miao language, and “Chaye Po” is a refined version of “Zaye Po” *(meaning “mixed-leaf slope”)*. This model reveals a “cultural borrowing” mechanism in which marginalized groups tend to use “cha” (tea), a mainstream cultural symbol with high recognizability and positive connotations, to rename places, thereby achieving a certain degree of cultural integration and identity reshaping. After all, in the process of transliterating ethnic languages and beautifying dialects through homophony, there are quite a few words that have the same pronunciation as the original words. Moreover, the original words reflect the local topographical features and cultural background to a certain extent. Transforming them into the word “tea” (cha) is a concrete manifestation of the potential influence of the tea industry and tea culture.

### Tea industry elements form the economic motivations

As a key driver of social development, the economy profoundly influences geographical naming conventions and renaming practices [73]. Hunan’s tea industry has a long legacy vividly reflected in its nomenclature. The analysis shows that the collected tea-related toponyms not only record individual tea-related economic activities, but also fully reflect the spatial layout of the entire tea industry chain in traditional society, from production and processing to logistics, trade, and end consumption. Toponym texts within the main category of tea industry reveal how economic functions influence the formation and naming of settlements. Through systematic code analysis, tea-related economic activities identified in local toponym texts, namely “making tea-related utensils,” “tea dealer track,” “tea trading business,” “running teahouses,” and “tea production”—are classified as components of the tea industry.

(1) The two subcategories “making tea-related utensils” and “tea production” reflect the specialized division of tea-related labor. The relevant tea-related toponym texts demonstrate the specialization and agglomeration effect of tea industry, which belong to economic activities at the production and processing stages. Toponyms such as “Chabei Ao” and “Chawu in Nanshan Village” function as identifiers of tea industry clusters formed in specific regions based on their resource endowments or technological advantages. The former is named from the fact that “before the Qing Dynasty, this place produced clay teacups,” while the latter is named after a house used for frying tea leaves. These toponyms are identifiers for tea industry clusters formed in specific regions based on their resource endowments or technological advantages. This naming mechanism emphasizes the professional identity of specific villages, making them specialized nodes in the regional economic network. (2) The toponym texts in the two subcategories “tea dealer track” and “tea trading business” reflect economic activities in the logistics and trade links of the tea industry, revealing the shaping of spatial structure through the circulation of tea industry. These are specific manifestations of the nodalization of the tea industry trade network. For instance, Chabu Zu of Zhiinan Village derives its name from its former tea-loading docks, highlighting the region’s pivotal role as a trading hub. Additionally, Chaweizi is a village whose tea sales have enriched the lives of its residents. These toponyms indicate that the location was, or still is, a key logistics or trade node in the regional tea trade network. It is evident that toponyms not only indicate location, but also record the trajectory of capital flows and the prosperity of the market. (3) The subcategory “running teahouses” demonstrates the spatial distribution characteristics of the tertiary industry and reflects the service stratification of tea-related consumption spaces. The analysis revealed that toponyms in this category exhibit a significant functional stratification, ranging from roadside rest stops that meet basic physiological needs. For example, Chapuzi derives its name from teahouses established to provide travelers with refreshments and rest, known as the thirst-quenching type. Elevated to public spaces with social functions, Chatingzi derives its name from vendors who sold tea for rest and leisure, referred to as tea-drinking-type teahouses. As venues hosting high-end cultural events, both Dengtangcunshangcha Zu and Dengtangcunxiacha Zu derive their toponyms from local teahouses, where people often gathered to discuss tea culture and philosophy, termed the tea-tasting type. As suitable venues for various tea-related consumption activities, Chaxinwu derives its name from a teahouse offering tea consumption and supplementary services, classified as comprehensive teahouses. This stratification reflects how tea-related economic activities meet the consumption needs of different social classes by providing varying levels of services, thereby reconstructing public living spaces in related settlements.

### Tea activity elements mirror the living habits

Tea activity refers to daily practices that have cultural significance [74], reflecting the core values embedded in people’s lives through tea-related customs and embodying the integration of material and spiritual pursuits [75]. Tea picking, tea processing, tea brewing, and tea tasting are all specific manifestations of tea-related activities. Chronologically, pre-tea activities include labor-intensive processes from planting to production, while post-tea activities involve experiential stages from brewing to savoring. Code analysis of Hunan’s tea-related toponyms identified tea-picking activities, tea-drinking utensils, and tea-drinking activities as key elements closely tied to residents’ daily lives, mirroring their living habits.

(1) The subcategory “tea-picking activities” originates from residents’ concrete behaviors, identity markers, and supporting infrastructure, forming the core of pre-tea activities independent of economic motives. The term Jicha Po *(tea storage slope)* refers to temporary tea storage areas that capture the essence of local harvesting practices. Toponyms such as Chanong *(tea farmers)* are derived from the convergence of tea-picking activities and community identity. Settlements referred to as Chating derive their names from rest pavilions for tea pickers, with such facilities providing logistical support. These toponyms are not arbitrary designations, but rather the result of workers’ tea-related physical practices, such as picking, carrying, and resting, which use toponyms to legitimize and solidify the “production space.” (2) The subcategory “tea-drinking utensils,” represented by the character “cha” (tea) in toponyms, typically denotes specialized vessels with distinctive shapes or materials and has an indicative function. Tea basins are predominantly wooden or sometimes stone, light-bottomed containers used to hold teapots, cups, tea sets, tea pets, and tea snacks. The toponym Chapengli derives its name from two stone tea basins in the settlement, reflecting local living conditions through unique material choices. Because of the unique nature of its materials, this special tea set transcends its practical functions and is transformed into a distinctive geographical symbol for identifying locations. It is evident that within the context of tea culture, microscopic objects can be symbolized and thus represent macroscopic geographical spaces, reflecting the close symbiotic relationship between objects and people. (3) The subcategory “tea-drinking activities” in toponyms originates from tea consumption practices. Through code analysis and Maslow’s Hierarchy of Needs [76], these activities are categorized into 1) physiological needs (quenching thirst), 2) safety needs (safe shelter for drinking tea and resting), 3) love and belonging needs (the preference for tea drinking and the sense of place generated by tea culture), 4) self-esteem needs (the need for respect through free tea), and 5) self-actualization needs (drinking tea and writing poetry for self-expression). Zhuozishicha Ting derives its name from a local tea stall that provides shelter for travelers. Moreover, Chayuan Zu in Yonghe Village derives its name from a teahouse that meets basic hydration needs. The “cha” (tea) in Chashui Ao reflects locals’ drinking habits and regional identity. Chating Pu derives its name from an old couple who used to offer pedestrians free tea in the pavilion and were respected by those pedestrians. The term “Laochating” *(old teahouse)* in Laochating Yuanzi refers to a pavilion where literati and scholars used to drink tea, compose poems, and play chess for self-expression.

### Tea polity elements incarnate the social backgrounds

Tea polity, also known as tea regulations, refers to historical government legislation governing the tea industry, including the establishment of tea management institutions, production, transportation, sales, and taxation [77]. In modern times, tea polity has been integrated into economic law and no longer exists independently. However, remnants of tea polity culture remain in tea-related toponyms, forming an essential part of tea culture. Through code analysis, “taxation and tribute” and “posthouse role” are categorized as tea polity, which partially describes the social context behind the formation and use of these toponyms.

(1) “Taxation and tribute” are government actions referring to tea culture-related elements in toponyms originating from government-imposed requirements, primarily taxation and tribute systems. Chaguan Zu collected water transport taxes on tea routes, serving as a concrete manifestation of government enforcement through taxation. Moreover, Guangcha Yuan refers to urban settlements supplying tea to imperial courts, representing local governments’ submission of tea as tribute. (2) “Posthouse role” denotes “cha” (tea)-related landmarks such as tea pavilions, teahouses, and tea shops, typically located at strategic points such as official roads, transportation hubs, or junctions, carrying political significance. For example, Chatingzi in Shichong Village originated from Ming Dynasty tea rest stations along official roads; Charenli is an ancient tea pavilion that served travelers on the Yunnan-Guizhou trade route, while Chating Pu, rumored to be a teahouse station at the intersection between Gaozhou and Yatian, was built for travelers’ convenience.

### Tea shape elements are the extension factors

The integration of regional culture into toponyms through metaphorical and analogical approaches is a common phenomenon in toponymy. During code analysis, numerous toponyms derived from geographical similarities were identified, whether through visual morphological resemblance or gustatory similarity. This study extracted and summarized tea culture elements in such toponym texts as “tea shape,” defined as a geographical metaphor and cognitive projection based on tea culture schemas. The term “tea shape” does not refer to the biological form of tea leaves, but rather to the process by which local residents, using familiar tea culture elements as reference points and employing techniques such as metaphor, analogy, and synesthesia, name and reconstruct the meaning of the natural geographical environment. Based on textual analysis and Lu Yu’s definition in *The Classic of Tea IV*, all tools used for roasting, grinding, boiling, drinking, and storing tea that contribute to the appreciation and refinement of tea and possess spiritual attributes are defined as tea utensils. Other tools, including those for picking, processing, and brewing tea, are defined as tea ware. The initial concept was further refined to obtain: terrain like tea utensils, water tasting like tea, terrain like tea-leaf, tea-colored soil or water, are categorized as tea shape elements. This category bridges natural and cultural dimensions, ingeniously integrating environmental characteristics into cultural attributes through geographical similarities, forming an extension of the structure of tea culture in toponyms. This reflects the extension of tea culture from “material practice” to “aesthetic perception.”

(1) “Terrain like tea utensils” refers to topographical features resembling specific tea utensils, including the terrain is similar to a tea-related utensil, the ground is similar to tea-related utensils, and the terrain is similar to tea ware. Specifically, the terrain is similar to a tea-related utensil, indicating topographical features similar to certain tea utensils. Textual analysis revealed that, in Hunan Province’s tea-related toponyms, this category primarily focuses on tea brewing, drinking, and storage. The name Chadouchong derives its name from terrain resembling a tea brewing funnel, often placed at the mouth of a teapot to prevent tea leaves from spilling out during brewing. It is a specific manifestation of tea brewing utensils. Chapo Dong derives its name from cup-shaped formations, representing tea-drinking utensils. Additionally, Chahuchong and Chapen derive their names from pot and basin shapes, respectively, indicating storage utensils. Similarly, “the ground is similar to tea-related utensils” refers to the presence of land features in a settlement that resemble the shape of a certain type of tea utensil. These land features include ponds, fields, courtyards, and temples, with ponds being the most numerous. The corresponding tea utensils include tea trays, teapots, tea bottles, and teacups, with tea trays being the most numerous. Chapang Chang, Chahu Tang, Chapingjin, and Chabei Tang were named after the shape of the pond in the residential area, which resembles a tea tray, teapot, teapot, and teacup. Chaping An features fields resembling tea trays, whereas Chaping Ao has a courtyard shaped like a tea tray. Matou Village’s Chapang An derives its name from a Qing Dynasty nunnery shaped like a tea tray. “The terrain is similar to tea ware” refers to terrain resembling specific tea-making ware, such as tea stalks or tea roasters, which relate to harvesting, picking, processing, and preparation. Chadou Po derives its name from terrain resembling a tea stalk during picking, symbolizing harvesting. Moreover, Chabeichong was named after terrain resembling a tool used in tea processing and roasting. (2) “Water tasting like tea” refers to well or spring water that tastes like tea, reflecting flavor similarities between water and tea. Chajingyi derives its name from locally sweet well water that tastes like tea, while Chadang derives its name from sweet, tea-like spring water in the local mountain. (3) “Terrain shaped like a tea leaf” describes topography resembling individual or stacked tea leaves. The former, exemplified by Chayechong*,* resembles a green tea leaf in the terrain of the settlement. The latter, exemplified by Chaduochong, features mountain shapes resembling stacked tea leaves. (4) “Tea-colored soil or water” refers to soil or water bodies in settlements displaying tea-brown hues. Chani Pang in Dayun Village derives its name from tea-colored earth, whereas Chajingchong derives its name from tea-green water in a well. Additionally, Chaxi derives its name from a stream flowing with the vibrant green hue of spring tea leaves. These categories represent the morphological simulation of material culture and the spatial expression of sensory synesthesia, reflecting tea culture’s deep involvement in spatial identity.

## Discussion and implications

### Discussion

**Fig 2:**
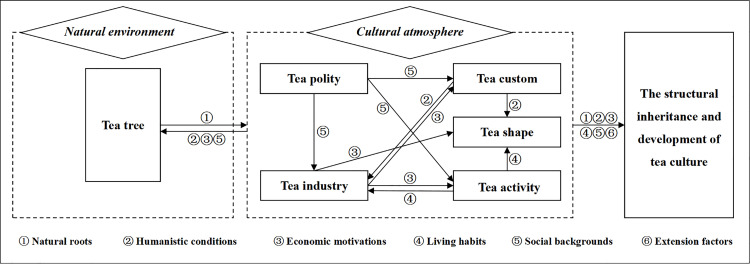
Tea culture structure and its internal relationship link.

Grounded theory analysis of tea-related toponym texts in Hunan Province reveals the cultural essence of tea traditions. Adopting a bottom-up approach leads to the emergence of six core categories, namely tea tree, tea custom, tea industry, tea activity, tea polity, and tea shape. This study reveals intrinsic connections among these categories, enabling the establishment of logical relationships across different dimensions and frameworks (Fig 2). The structural framework of tea culture and its internal relational network effectively explain its formation mechanisms, the interconnections between elements, and the specific drivers behind its structured inheritance and development.

### The internal structure of tea culture has formed two mutually influential unit systems

The complex layers of South Korean tea culture constructed by Kim et al. specifically include tea texts, sets, ceremonies, and places [37]. The authors place these four elements of tea culture in the same dimension. Botejue proposed that the connotation of Sri Lankan tea culture specifically encompasses material culture related to tea, linguistic practices, behavioral patterns, and the political influence of tea culture [39]. This enriches the specific content of the connotations of tea culture. However, relevant studies have not explored the internal relationships within the tea culture structure. In addressing this gap, the current study not only enriches the elements of tea culture, but also further explores the multiple cross-relationships among these elements. From a holistic perspective, tea culture is the result of the interaction between the natural environment and the cultural atmosphere, with the two influencing each other. Within this holistic unit, tea tree is the core of the natural environment, while tea custom, tea industry, tea activity, tea polity, and tea shape form the cultural atmosphere. From the perspective of internal structural units, six elements including tea tree, tea custom, tea industry, tea activity, tea polity, and tea shape form the “effect-feedback” mutual influence pattern through synergistic interactions, ultimately propelling the structured inheritance and evolution of tea culture. Lichun et al. [78] used a cultural gene map to explore the complex structure of Anhua Dark Tea in terms of its material and spiritual culture. This suggests that tea does not comprise a single structure and that the tea culture formed around it is complex and layered. The inheritance and development of tea culture is not a linear process, but rather exhibits structural and comprehensive characteristics.

### Tea tree forms the core of the natural environment unit in the tea culture structure

Without informing cultural models with deepening knowledge of the structures and processes of the mind, cultural analyses may be reduced to mere literary exercises [79]. The same holds true for tea culture research, which should examine the complex conceptual relationships among tea culture’s constituent elements. Based on grounded theory analysis, this study finds that tea tree elements, as the intrinsic root of the tea culture structure, have a close logical mapping relationship with elements related to tea custom, tea industry, tea activity, tea polity, and tea shape (Fig 2). On the one hand, the pathways through which tea tree elements interact involve tea custom, tea industry, tea activity, tea polity, and tea shape. On the other hand, elements of the tea industry, tea polity, and tea custom provide feedback pathways to tea tree elements. This is consistent with theories of logical relationships among cultural elements and components [80]. This pattern of influence is mainly reflected in three aspects of the toponym texts: economy, politics, and local culture (Fig 2).

Tea industry elements and tea tree elements exhibit mutual influence based on their economic functions. The toponym texts show that the prosperity of the tea industry is often accompanied by the expansion and consolidation of tea tree planting spaces. This does not imply that toponym texts directly demonstrate causal growth in economic data, but rather reflects how the concept of “B_10_: Tea trading business” strengthens residents’ emphasis on and protection of “B_2_: Tea planting and cultivation” [81]. For example, some settlements with “Chayuan” in their toponyms, even if destroyed during historical changes, are often restored in subsequent agricultural development because of their potential economic value. This, to some extent, demonstrates that the “tea industry” has given “tea tree” an economic rationale, thereby strengthening the survival of “tea tree” elements in space.

Tea polity provides a political framework for maintaining tea tree [82,83]. There is a significant structural link between “tea polity” (such as “B_16_: Taxation and tribute”) and “tea tree” (such as “B_2_: Tea planting and cultivation”). Toponyms such as “Guancha Yuan” indicate that government administrative needs related to tea directly intervene in specific production spaces, making “B_2_: Tea planting and cultivation” not only an agricultural activity but also a political task. This connection reveals how political forces, by establishing the legitimacy and necessity of planting, conceptually and spatially stabilize the existence and development of “tea tree.”

Tea custom is the cultural foundation in which tea tree elements are deeply rooted in local society. There is an internalized relationship between “tea custom” (such as “B_4_: Faith and custom”) and “tea tree” (such as “B_2_: Tea planting and cultivation”) as a way of life. Terms such as Chashui Ao and Qingcharong reflect local residents’ habit of “not being able to go a day without tea.” This custom does not unilaterally influence tea cultivation but rather creates sustained social demand, making tea cultivation an indispensable part of the local way of life. In essence, “tea custom” provides a socio-psychological basis for the recognition of “tea tree,” and the two are mutually reinforcing within the local cultural system.

### Tea custom, tea industry, tea activity, tea polity, and tea shape form the cultural atmosphere unit of tea culture structure

Whitfield believed that clarifying the social positioning of tea culture was beneficial to its development [38]. Similarly, clarifying the conceptual positioning and interaction logic of each element within the structure of tea culture is key to theoretical construction. IIn the structure of tea culture, the cultural atmosphere comprises tea custom, tea industry, tea activity, tea polity, and tea shape. Tea custom reflects humanistic conditions, tea industry provides economic motivation, tea activity mirrors living habits, tea polity describes social backgrounds, and tea shape extends the aforementioned cultural connotations. There is also an “effect-feedback” mutual influence among these categories.

First, tea industry has pathways of influence on tea custom, tea activity, and tea shape, with comprehensive characteristics. In other words, various aspects of tea elements interact with various aspects of tea custom, activity, and shape. This is consistent with a comprehensive view of tea culture structure [79].

Next in importance, tea custom and tea activity provide feedback pathways to tea industry. This is mainly reflected in the fact that “B_4_: Faith and custom” and “B_15_: Tea drinking activities” can drive “B_10_: Tea trading business” and “B_11_: Running teahouses.” It is worth noting that this is not a mechanical feedback loop but rather a manifestation of cultural demand that constructs the legitimacy of economic supply. Specifically, “tea custom” and “tea activity” create stable social consumption scenarios. These cultural practices provide a social foundation and market logic for “B_10_: Tea trading business” and “B_11_: Running teahouses.” This foundation conceptually reflects the projection of “demand pulls supply” in economics onto cultural and geographical space [84].

Furthermore, tea polity has pathways of influence on tea industry, tea custom, and tea activity. Evidence from toponym texts shows that tea polity, especially the “B_17_: Posthouse role” in this field, serves as an institutionalized infrastructure that provides the necessary physical space and facilitating conditions for the development of tea industry, tea custom, and tea activity. In grounded theory textual analysis, tea polity functions more as a top-down external constraint or enabling force, with no clear bottom-up reconstruction. This reflects the specific impact of political power on spatial production during historical periods.

Finally, tea custom and tea activity have a unidirectional effect on tea shape, mainly because the “B_4_: Faith and custom” in tea custom and the “B_15_: Tea drinking activities” in tea activity have a potential influence on the formation of tea shape elements.

In a word, at the cultural atmosphere level of the structure of tea culture, the participation of human behavior is extensive and direct, naturally reflecting the interactive relationship between “human-tea-land.”

### Theoretical implications

This study makes the following contributions to the literature.

First, it explores the structural system of tea culture through toponym texts, integrating tea studies and toponymy and enriching the theoretical perspective and knowledge system of tea culture research. Existing studies mostly follow the classic paradigm of cultural sociology, examining tea culture from the perspective of social phenomena related to tea and focusing on macro-level aspects such as cultural and literary expression, summarizing tea culture structure as a “material-spirit” binary structure [53], or a four-level structure comprising “material-institution-spiritual-behavioral” dimensions [54], etc. This study, through a systematic deconstruction of the historical and geographical imprint of “tea-related toponyms,” establishes the six core categories of tea culture structure, including tea tree, customs, industry, activities, polity, and shape. Simultaneously, by connecting these categories through “storylines,” a tea culture structure that covers multiple dimensions including “material-spiritual-industrial-institutional-behavioral-cognitive,” is ultimately constructed. This finding not only reveals the unique value of toponym texts as “living fossils” of tea culture, but also provides a more diverse and specific new cognitive framework for understanding tea culture structure and the interrelationships among its internal elements across spatial distribution and social function, thereby expanding the theoretical boundaries of tea culture research.

Second, by exploring the structural elements of tea culture, this research examines how these elements mutually influence each other, thereby enhancing the explanatory power of the theoretical framework. While existing studies have constructed diverse structural systems for tea culture, they have often focused on the independent interpretation or parallel description of single elements [53,54,85,86]. This study employs grounded theory to construct a tea culture structure from tea-related toponym texts and explores the relationships among the elements within the tea culture structure from the perspective of connections. The findings indicate that there is an “effect-feedback” mutually influential relationship among the various elements that constitute the tea culture structure, providing theoretical support for a deeper understanding of the evolutionary patterns and inheritance logic of tea culture.

Third, this study examines the structure of tea culture within the context of human-land relations, further clarifying the interactive logic between “human-tea-land.” This finding transcends existing research that statically deconstructs tea culture from different perspectives, such as cultural resource types [87], value core [88,89], experiential perception [90], and artifact design [91]. In doing so, it clarifies the dynamic evolution of tea culture within toponymic space. In terms of patterns, the six elements of tea culture structure show a progressive distribution from “natural materials” to “social and cultural elements,” reflecting the evolution of human civilization from adaptation to the natural environment to the creation of a cultural atmosphere. From a mechanistic perspective, tea culture reflected in tea-related toponyms forms a structured framework of “material identification-spiritual symbolism-industrial development-institutional governance-behavioral practice-cognitive projection.” This finding enables analysis how “tea” serves as a medium connecting “human” and “land,” and provides a novel theoretical explanation for understanding the spatial construction and structural development of regional cultural landscapes.

### Practical implications

The findings of this study not only have theoretical value, but also provide specific path guidance for cultural heritage protection and inheritance, industrial development, industrial integration, and industrial innovation at the practical level.

First, this work provides decision-making support for the systematic protection and structured inheritance of tea culture and toponym cultural heritage. Research verifies that, in areas with developed tea culture, tea culture and toponym culture are mutually related, and toponyms are valuable texts for exploring and preserving local culture, including tea culture. The research results can directly aid cultural heritage protection by guiding relevant departments in conducting systematic surveys, identification, and digital archiving of tea-related toponyms, while preventing the loss of cultural memory. Simultaneously, the “complexity” and “regionality” of the tea structure suggest that protection policies should avoid “fragmentation” and rather, adopt a holistic protection strategy. While protecting the natural foundation of the “tea tree,” policies should also coordinate the preservation of related folk customs, skills, historical relics and cultural landscapes to form a sustainable system of living inheritance.

Second, the findings increase the added value of tea products and inject cultural value into the tea industry’s value chain and brand-building. This study clarifies the “effect-feedback” mechanism between tea cultural elements and economic activities. Practically, tea companies can leverage this model to incorporate the profound cultural connotations of toponyms into brand narratives and product designs, thereby enhancing brand premium capabilities. Simultaneously, by developing creative tea utensils, tea snacks, and cultural and creative derivatives that are closely integrated with cultural elements, it is possible to transcend the physical limitations of tea as an agricultural product, expand the boundaries of the industry, and achieve a leap from “product output” to “culture and lifestyle output,” thereby enhancing core competitiveness in domestic and international markets.

Third, it provides new approaches for the in-depth integration of culture and tourism, as well as the development of regional brands. The six-element structure identified in this research provides a systematic framework for the development of tea culture tourism products. In destinations with a strong tea culture, practitioners can use this model to thoroughly explore and integrate local tea custom (such as tea ceremonies and festivals), tea activity (such as tea picking and tea-making experiences), tea polity (such as the history of the Ancient Tea Horse Road), and tea shape (such as landscapes named after tea). Moving beyond simple experience of tea garden sightseeing and tea tasting, they can design immersive and narrative cultural tourism routes and activities, thereby achieving a transformation and upgrade from “selling resources” to “selling culture,” effectively enhancing the added value and unique charm of related tourism products.

Fourth, this study provides insights into the interpretation of tea culture connotations in tea-producing regions of countries such as China, the United Kingdom, Japan, South Korea, and Turkey. Although these countries differ in their natural endowments and social customs, they share common characteristics in the connotations of tea culture and its generation logic, such as the interaction and evolution of elements, like the development of tea-related activities, the development of the tea industry, and the formation of tea customs [92–95].

### Limitations and further work

Generally, this study is innovative in both perspective and methodology. However, it has certain limitations. First, while grounded theory strictly follows step-by-step logic and research team members conduct coding separately to minimize subjective influence, the method as a qualitative research approach cannot completely eliminate subjective factors. Second, this study is still based on data from a single region (Hunan Province). Although the constructed tea culture framework is derived from extensive data on tea-related toponyms across Hunan Province through systematic conceptualization and iterative discussion, it lacks more thorough and comprehensive field investigation. This may result in discrepancies between the interpretation of tea culture in toponyms and actual conditions. Although tea-related toponyms in Hunan are representative, they do not fully capture other regions or the entire country, which limits the generalizability of the conclusions. Third, toponym texts have descriptive characteristics that constrain their use as qualitative data in grounded theory. As secondary data, toponym texts do not allow for immediate follow-up questioning, unlike interview-based data. Future research should conduct large-scale field surveys to collect tea-related toponym data from other regions to improve the saturation testing of grounded theory. Additional methods should be employed to verify textual accuracy, develop corresponding measurement scales, and apply quantitative approaches, such as structural equation modeling, to further validate the framework and establish a more rigorous and comprehensive tea culture structural system.

## Supporting information

S1 File Open coding of tea culture. (PDF)

S2 File Axial coding of tea culture. (PDF)

S3 File Theoretical coding of tea culture. (PDF)
