# Supplementary material for: The intrinsic structure and interrelations of tea culture constructed from tea-related toponym texts: Evidence from China
Source: PLoS One. 2026 Apr 17;21(4):e0347109. doi: 10.1371/journal.pone.0347109 (PMC13089723; doi:10.1371/journal.pone.0347109)
Supplement: S1 File — (PDF) [file pone.0347109.s001.pdf]

Open coding of tea culture

Primitive statements (example)	Label extraction	Conceptualization (A_n)	Subcategory (B_n)
A toponym was named because there was a tea tree about 4 meters high in this place before.	A 4-meter tea tree	**A_2:** There was a tall tea tree	**B_1:** Lonely ancient tea trees
There was a piece of dry land where tea was grown and the land contract system was implemented. It is allocated to seven or eight groups. Since the early 1980s, houses have been built to form a residential area.	A piece of dry land; Grow tea	**A_4:** Grow all kinds of tea	**B_2:** Tea planting and cultivation
A toponym was named because it had a large tea plantation and was always rich in tea production.	The original large tea plantation; Rich in tea production	**A_9:** Tea plantations in the historical period	**B_3:** Tea tree plantations
There was a large stone-paved path in the middle of the courtyard, which passes through the middle of the pavilion. On the Dragon Boat Festival, someone brews tea.	Someone brews tea on the Dragon Boat Festival; The pavilion	**A_{12}:** Local customs are related to tea	**B_4:** Faith and custom
In the early years, there was a hermitage dedicated to the tea god.	A hermitage; Dedicated to the tea god	**A_{14}:** A temple was built to worship the god of tea	
In 1982, the place was divided into groups. The adjacent tea plantations were named a toponym, symbolizing that people's life is like spring tea leaves, red and lively.	Adjacent tea plantations; It signifies a prosperous life	**A_{15}:** The good meaning of tea without worry	**B_5:** Beautiful metaphor
Legend has it that a man surnamed Li lived by a hillside at the foot of Huangmao Mountain. He planted several tea seedlings, but while others withered, one cluster in the center grew larger	Divine guidance from Guanyin Bodhisattva; Tea could cure all	**A_{21}:** The legend of tea curing diseases	**B_6:** Allusions and legends

Primitive statements (example)	Label extraction	Conceptualization (A_n)	Subcategory (B_n)
than a blue plate. The kind-hearted old man and his neighbor were so compassionate that when his wife fell gravely ill, she received divine guidance from Guanyin Bodhisattva. She cured her illness with tea leaves, and this miraculous plant grew to produce tea that could cure all diseases. As this area became a crucial route between Longwan Bridge and Yueyang City and gained fame for its tea.	diseases; A crucial route		
Legend has it that long ago, a tea-picking girl went to the river in front of the village to wade across but drowned and was later deified, hence the name.	Tea-picking girl; The girl becomes immortal	**A_{23}:** The story of the tea-picking girl	
"Da" is an adjective; "Cha Hu" is a Tujia word, and in the Tujia script, it is "Carhur." "Cha" is a phonetic variation of another word with a similar pronunciation, meaning "good"; "Hu" is translated as "valley" or "ditch" in Chinese.	Tujia phonetic changes; Conversion between different languages	**A_{24}:** The languages of ethnic minorities are translated into Chinese according to their pronunciation	**B_7:** Language and culture
The toponym originates from the Miao language. "Cha" refers to Han Chinese, while "dong" means a hollow or pit. In ancient times, this area was inhabited by Han Chinese.	Derived from Miao language toponym; Conversion between different languages		
It is said that this place was originally the camp of Huang Chao's troops, so it was called a toponym and later it was renamed another toponym owing to phonetic similarity.	The place where troops set up camp; Phonetic similarity	**A_{25}:** Dialect homophonic beautification	
The settlement was named after its location near	Near the tea	**A_{44}:** A tea factory was	**B_{12}:** Tea

Primitive statements (example)	Label extraction	Conceptualization (A_n)	Subcategory (B_n)
the former tea processing factory.	processing factory	built to make tea	production
There was a house here that specialized in frying tea leaves, hence the name "Chawu." "Chawu" is a house used for frying tea leaves.	A house specialized in frying tea leaves	**A_{46}:** There are tea processing places	
In the early days after liberation, this place was mainly used for tea picking and was named the toponym.	Tea picking is the main agricultural activity	**A_{47}:** The agricultural activity of picking tea	**B_{13}:** Tea-picking activities
Because many tea trees were planted on the hillside, tea farmers settled here.	Many tea trees were planted; Tea farmers settled here	**A_{48}:** Where tea farmers live	
The name refers to a town or settlement that originally supplied tea to the imperial court. Because the tea grown here was once tribute tea.	Originally supplied tea to the imperial court; Tribute tea	**A_{56}:** During the historical period, tea was supplied to the imperial court	**B_{16}:** Taxation and tribute
The toponym is derived from the name of a well. There is a well in the valley whose water is tea-colored, hence the name.	The well water was tea-colored; Derived toponym	**A_{70}:** Some wells have tea-colored water	**B_{21}:** Tea-colored soil or water

Tips: the content in the table is examples of open coding for tea culture in tea-related toponym texts.
