# Supplementary material for: The intrinsic structure and interrelations of tea culture constructed from tea-related toponym texts: Evidence from China
Source: PLoS One. 2026 Apr 17;21(4):e0347109. doi: 10.1371/journal.pone.0347109 (PMC13089723; doi:10.1371/journal.pone.0347109)
Supplement: S2 File — (PDF) [file pone.0347109.s002.pdf]

Axial coding of tea culture

number	fundamental category	Corresponding subcategory
**C_1**	Tea tree	**B_1:** Lonely ancient tea trees; **B_2:** Tea planting and cultivation; **B_3:** Tea tree plantations
**C_2**	Tea custom	**B_4:** Faith and custom; **B_5:** Beautiful metaphor; **B_6:** Allusions and legends; **B_7:** Language and culture
**C_3**	Tea industry	**B_8:** Making tea-related utensils; **B_9:** Tea dealer track; **B_{10}:** Tea trading business; **B_{11}:** Running teahouses; **B_{12}:** Tea production
**C_4**	Tea activity	**B_{13}:** Tea-picking activities; **B_{14}:** Tea drinking utensils; **B_{15}:** Tea drinking activities
**C_5**	Tea polity	**B_{16}:** Taxation and tribute; **B_{17}:** Posthouse role
**C_6**	Tea shape	**B_{18}:** Terrain like tea utensils; **B_{19}:** Water tasting like tea; **B_{20}:** Terrain like tea-leaf; **B_{21}:** Tea-colored soil or water
