# Supplementary material for: The intrinsic structure and interrelations of tea culture constructed from tea-related toponym texts: Evidence from China
Source: PLoS One. 2026 Apr 17;21(4):e0347109. doi: 10.1371/journal.pone.0347109 (PMC13089723; doi:10.1371/journal.pone.0347109)
Supplement: S3 File — (PDF) [file pone.0347109.s003.pdf]

Theoretical coding of tea culture

Core Scope	Subcategory	Initial Concepts
C_1: Tea Tree	B_1: Lonely ancient tea trees	A_1: There are ancient tea trees that are many years old
		A_2: There was a tall tea tree
		A_3: The village above a tea tree
	B_2: Tea planting and cultivation	A_4: Grow all kinds of tea
		A_5: The village used to grow tea in historical times
		A_6: Artificially cultivated tea
		A_7: Existing land for specialized tea
		A_8: Abundant tea production
	B_3: Tea tree plantations	A_9: Tea plantations in the historical period
		A_{10}: Tea trees grow in gardens
		A_{11}: Wild tea plants are often grown in gardens
C_2: Tea Custom	B_4: Faith and custom	A_{12}: Local customs are related to tea
		A_{13}: Etiquette of drinking tea
		A_{14}: A temple was built to worship the god of tea
	B_5: Beautiful metaphor	A_{15}: The good meaning of tea without worry
		A_{16}: The beautiful meaning of tea fragrance
		A_{17}: The good symbol of tea-leaf
	B_6: Allusions and legends	A_{18}: Legend of the magical tea tree
		A_{19}: An allusion to the origin of tea culture
		A_{20}: Famous people and tea legends
		A_{21}: The legend of tea curing diseases
		A_{22}: The legend of tea plantation
		A_{23}: The story of the tea-picking girl

Core Scope	Subcategory	Initial Concepts
	B_7: Language and culture	A_{24}: The languages of ethnic minorities are translated into Chinese according to their pronunciation
		A_{25}: Dialect homophonic beautification
		A_{26}: Local names are special references
		A_{27}: People with the surname “Cha” live here
C_3: Tea Industry	B_8: Making tea-related utensils	A_{28}: Production of teacup clay
		A_{29}: Some people know how to make tea trays
		A_{30}: There are well-known masters of handmade tea utensils
	B_9: Tea dealer track	A_{31}: There used to be a dock for loading and unloading tea
		A_{32}: There is a tea pavilion on the way to the customer
		A_{33}: A pavilion built by a tea merchant
	B_{10}: Tea trading business	A_{34}: Wholesale or retail tea
		A_{35}: There are tea shops for buying and selling
		A_{36}: Tea houses that buy tea
		A_{37}: The local economy depends on tea
		A_{38}: The tea industry flourished in history
	B_{11}: Running teahouses	A_{39}: Open teahouses in local areas
		A_{40}: Building a teahouse
		A_{41}: Build a pavilion for opening a teahouse for pilgrims to drink tea
		A_{42}: There are tea stalls
		A_{43}: There was a teahouse
	B_{12}: Tea production	A_{44}: A tea factory was built to make tea
		A_{45}: There are tea making companies
		A_{46}: There are tea processing places
C_4: Tea	B_{13}: Tea-picking	A_{47}: The agricultural activity of picking tea

Core Scope	Subcategory	Initial Concepts
Activity	activities	A_{48}: Where tea farmers live
		A_{49}: Build a pavilion for people to pick tea and rest
	B_{14}: Tea drinking utensils	A_{50}: The residential area has two stone tea basins
	B_{15}: Tea drinking activities	A_{51}: Locals like drinking tea
		A_{52}: There are tea tasting and tea culture propagating activities
		A_{53}: Scholars drink tea and compose poems
		A_{54}: There is a place to have tea and rest
		A_{55}: Tea culture flourished locally
C_5: Tea Polity	B_{16}: Taxation and tribute	A_{56}: During the historical period, tea was supplied to the imperial court
		A_{57}: There was a customs duty on tea transported by water
	B_{17}: Posthouse role	A_{58}: There are places for pedestrians to drink tea and rest
		A_{59}: A tea pavilion was built as a transportation station
C_6: Tea Shape	B_{18}: Terrain like tea utensils	A_{60}: The terrain is similar to a tea-related utensil
		A_{61}: The ground is similar to tea-related utensils
		A_{62}: The terrain is similar to tea ware
	B_{19}: Water tasting like tea	A_{63}: The water from the well is as sweet as tea
		A_{64}: Spring water is like tea
	B_{20}: Terrain like tea-leaf	A_{65}: The shape of the place resembles a tea leaf
		A_{66}: The terrain resembles a tea stack
		A_{67}: Terrain resembles a tea plantation
		A_{68}: There are small beaches like Chaping
	B_{21}: Tea-colored soil or water	A_{69}: The soil is tea-colored
		A_{70}: Some wells have tea-colored water
		A_{71}: The stream is as green as tea
